# Efficacy of different irrigation needles used in endodontics: an *in silico* and an *in vitro* investigation

**DOI:** 10.2340/biid.v12.45148

**Published:** 2025-12-17

**Authors:** Maulee Sheth, Ankit Arora, Sonali Kapoor, Balraj Shukla

**Affiliations:** a Department of Conservative Dentistry and Endodontics, Manubhai Patel Dental College and Hospital, Maharaja Krishnakumarsinhji Bhavnagar University, Vadodara, Gujarat, India; b Department of Pediatric and Preventive Dentistry, College of Dental Sciences and Research Centre, Gujarat University, Ahmedabad, Gujarat, India

**Keywords:** Endodontics, root canal preparation, root canal therapy, fluid dynamics, 3D printing, dental irrigation needle

## Abstract

**Objective:**

Irrigation is a clinical procedure in which needles of various designs, attached to syringes, are delivered by positive pressure to cleanse the root canals of infection-promoting agents. Despite being available in multiple designs and different materials, the irrigant does not necessarily reach every portion of the canal. This study compared four different needle designs in terms of depth of penetration of the needle (DOP), wear of the needle and fluid dynamics of the irrigant (FD).

**Methods:**

Stereolithography was used to manufacture four 3D-printed single-rooted mandibular premolars with double curvature. The four needle designs used were Group I (NiTi open-ended, notched needle tip), Group II (Stainless steel, single-sided vented needle tip), Group III (Stainless steel, double-ended needle tip), and Group IV (Soft propylene, multi-vented needle tip) (*n* = 10 in each group). After assigning each tooth to a group, a stereomicroscope was used to measure the DOP. Pre- and post-irrigation scanning electron microscopy images of three randomly chosen needles from each group helped qualitatively determine the wear. Later, particle image velocimetry (PIV) experiments for each of the four needle designs were subsequently compared with those obtained from computational fluid dynamics (CFD).

**Results:**

The open-ended syringe had the significantly lowest mean DOP as determined by one-way ANOVA and Tukey’s post hoc test. Wear was significantly lowest in the non-metallic syringes. The PIV and CFD analyses were in close agreement with each other. The non-metallic needle exhibited the highest pressure and axial velocity near the apex.

**Conclusion:**

The validated CFD models showed a greater canal coverage and irrigant flow from the non-metallic syringe in the double curvature root canal simulations; though with the highest risk of apical extrusion.

## Introduction

Irrigation in dentistry is an essential procedure performed during root canal treatment, wherein the root canals of the affected tooth are thoroughly cleansed and disinfected by the introduction of suitable irrigants [1]. These irrigants enter the root canals with the help of hypodermic needles of various designs attached to a syringe. The understanding of the kinematics of irrigants has thus been a core research topic, driven by the technological advancements of the 21st century. The factors associated with the irrigant (viscosity, velocity, pressure) and the needle (penetration depth, diameter, type, orientation of the needle bevel, canal diameter) govern the irrigant’s fluid dynamics (FD) [2, 3].

The most prevalent technique to deliver irrigants in the root canals has been through open-ended stainless steel needles. However, a gray area exists in terms of its effectiveness, based on the root canal coverage of the irrigants that can ensure adequate irrigant replacement, as well as the required effective shear stress to clean the canals. Root canals untouched by the irrigant can thus be prone to persistent or recurring infection [4, 5].

The rationale for our study was thus to computationally investigate the FD of irrigants when they are delivered through needles of different designs and materials in a validated double-curvature root canal model. Since open-ended needles are commonly used, we used its variant by opting for one that is made of NiTi. Owing to NiTi’s flexibility, we postulated it to be a better fit for double-curvature canals. Stainless steel needles with vents on either a single side or both sides in a double-curvature canal were chosen for their market prevalence. Finally, our fourth needle was the soft polypropylene needle, which is non-metallic compared to the other three syringes. Moreover, literature on the soft polypropylene needle’s flow characteristics in validated root canal models is sparse.

Experimental, theoretical, and computational means exist to understand an irrigant’s FD. Although Computational Fluid Dynamics (CFD) was introduced as a tool for engineers and industries, it has made remarkable inroads in the field of endodontics [6]. Through CFD, analysis of irrigation dynamics within various root canal anatomies (oval-shaped, straight, with apical delta, single curvature canals), through different designs of irrigant needles (bevelled, open-ended, closed-ended, notched, side-vented, multi-vented), at various working depths, and at varied flow rates have been reported [5–14]. The governing equations of physics and mathematics have validated these simulations of the irrigants. These equations are used to perform parametric investigations in validated root canal models for CFD. High-speed imaging and particle image velocimetry (PIV) are standard tools used to validate the CFD model [6].

Surface irregularities on either the internal or external diameter of the irrigation needle can affect the flow of the irrigant [15]. The challenge further intensifies in root canals that are S-shaped or have double curvature, where the irrigant flow can be compromised due to a shallower penetration depth [16–18]. The understanding of an irrigant’s FD in double curvature canals remains unexplored. Understanding irrigation dynamics in various simulations and environments is always beneficial, as it helps us enhance the effectiveness of irrigation based on its working mechanism and its approach when interacting with microbes. A relatively recent retrospective study has shown an association between complex anatomy and untouched canals as predictors for endodontic treatment failure following primary root canal treatment [19]. Thus, this study aimed to compare the irrigant flow through metallic and non-metallic syringes with different designs in double curvature canals.

Due to the variations of the needle geometries and materials used in the study, no assumptions were made to deem one design superior to another for a clinically successful flow of irrigant. To achieve our objective, we utilized 3D-printed tooth models that underwent *in vitro* and *in silico* analysis to assess the penetration depth of the needle, changes in wear on the needle, and flow characteristics of the irrigant. A key component of our study was the validation of the CFD model, which enabled us to gain a deeper understanding of irrigation dynamics in various simulations. The reporting of this study was guided by the Preferred Reporting Items for Laboratory Studies in Endodontology (PRILE) 2021.

## Materials and methods

An institutional ethics committee (MPDC_220/CONS-38/21) granted ethical clearance for the conduct of this research.

### Tooth models

**Figure 1:**
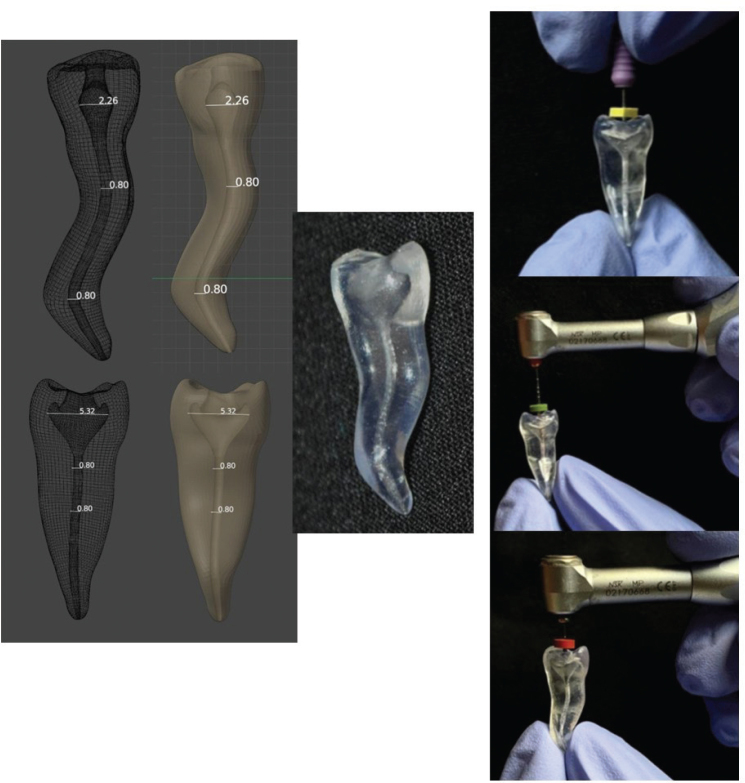
Computer-assisted design of the 3D printed tooth model. The instruments for biomechanical preparation were hand files, followed by the use of rotary files for enlargement of the canal till #30.04.

A stereolithography (STL) printer (Fab Pro 1000) from Invision Dental Models (Mumbai, India) created four 3D-printed tooth models made of polymethylmethacrylate. Model slicing of the output STL file resulted in the conversion of the 3D CAD model into multiple layers of 2D cross-sections. The length of each 3D-printed tooth was 21 mm, with two curvatures at 8 and 18 mm, measuring 19 and 28 degrees, respectively, as per Schneider’s method [20] (Figure 1).

### Sample distribution

**Figure 2:**
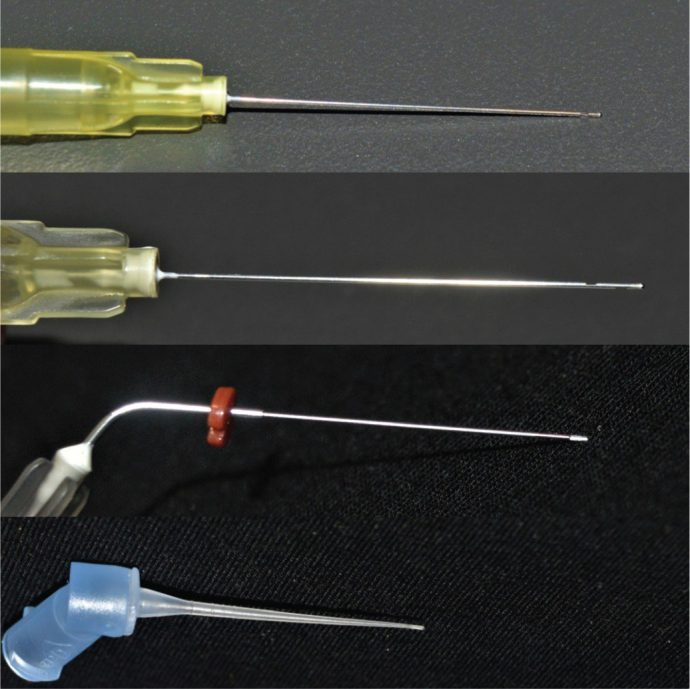
The needles used in this study (from top to bottom) – Stainless steel single-side vented needle, stainless steel double-side vented needle, NiTi open-ended notched needle, soft polypropylene multi-vented needle.

This study used a purposive sampling strategy. Each of the four prepared tooth models was assigned to one of the four groups in our study. Brand-new irrigation needles (attached to single-use irrigation syringes) were used in each group (*n* = 10 per group). These were as follows – Group 1: NiTi open-ended needle tip (NiTi SuperFlex, Vista Apex Dental Products, Wisconsin, USA), Group 2: Stainless Steel double-vented (SSDV) needle tip (Mirajet Endo Rinse, Hager Werken, Duisburg, Germany), Group 3: Stainless Steel single-vented (SSSV) needle tip (Neo Endo side vented needle, Orikam Healthcare Pvt Ltd., Gurgaon, India), and Group 4: Soft polypropylene back-to-back side vented close-ended needle tip (TruNatomy, Dentsply Sirona, Maillefer Instruments, Switzerland) (Figure 2).

### Depth of penetration, irrigation protocol, wear

All needles in their respective groups were passed within the tooth model’s canal until the maximum depth of penetration (DOP) (1.5 mm short of the bound position). The depth of needle placement was in accordance with previously recommended guidelines to minimize the risk of extruding apical debris [21, 22].

Two endodontists calibrated for this study performed the procedure. They were assigned five needles each in each group. A stereomicroscope at 100x magnification (Olympus Corporation: Model SZ 2-LGB) quantitatively measured the distance from the tip of each needle to the apex. The recorded results of DOP for each needle were tabulated in Microsoft Excel for statistical analysis. Before irrigation, three needles from each group were observed under a scanning electron microscope (SEM) at magnifications ranging from 200x to 2,000x (JEOL: JSM-638OLV) to record the pre-irrigation health of the needles. Gold sputtering of the non-metallic needle provided better visualization under SEM.

While performing the SEM, the researchers also found the internal and external diameters of each needle type. The endodontists were unaware of which of the three needles in each group were used for the SEM imaging. Two assistants were tasked with registering the 12 needles (three from each group) used in the SEM imaging.

Biomechanical preparation was done in all four tooth models using hand K-files (Mani Inc., Tochigi, Japan) and Edge Endo (X7 series) rotary file system (Flexicon, US Endodontics, Johnson City, USA). A master apical file of #30.04% was established. This enlargement of the canal has been recommended for curved canals as per a previous study that validated the claim by observing a decrease in apical pressure due to the irrigant’s dynamics [8] (Figure 1).

A 2.5 mL single-use irrigation syringe (Unolok, Hindustan Syringes & Medical Devices Ltd., India) facilitated copious irrigation with 5.25% sodium hypochlorite (Prime Dental Products Pvt. Ltd., India) and 0.9% saline (Aculife Healthcare Pvt. Ltd., India). The 10 needle tips of the respective groups were used for the final irrigation protocol using 5 mL of 5.25% sodium hypochlorite, followed by 0.9% saline solution. The rate of irrigation was 1 mL/min, for each solution. After every successive irrigation performed by the needles in each of their respective groups, the canals were dried using paper points and sterilized with 95% ethanol.

After irrigation, the 12 needles that had undergone SEM imaging before use were now re-examined to obtain post-irrigation SEMs. The wear of the irrigation needles were re-examined under the same magnification. An expert in metallurgy and an endodontist prepared two separate reports based on their observations of the wear in each of these needles before and after irrigation. A theme-based approach facilitated the making of qualitative observations from the images.

### Experimental setup for model validation of the double curvature canal

Since irrigants in a root canal are exemplary of a fluid in a velocity field, PIV was done for each needle design. Each needle design and the tooth model was considered as a dyad. The methodological protocol for PIV setup was similar for each of the needle-design-dyads. The tooth model was mounted on a luer-lock connector and positioned in front of an optical microscope (Standard variant of BX53M Microscope, Evident Scientific, Japan). The microscope was attached to a high-speed camera (Chronos 1.4, Kron Technologies, Canada), which was capable of recording over 40,400 frames per second. However, the images at this FPS were low in resolution, and thus we chose an FPS of 31,294 for better quality. A continuous cold light source was used to achieve better resolution in the imaging. A precision syringe pump was used for delivering the irrigants inside the canal. Each needle (attached to the syringe pump) was positioned at its mean DOP as derived from the statistical data analysis in Microsoft Excel.

The irrigant was delivered at a rate of 0.26 ml/s as per the recommended optimal speed of irrigant administration [21]. Neutrally buoyant tracer particles of 1.1g/cc density (MMSC, Maharashtra, India) were then introduced to allow the visualization of the irrigant’s flow. Since the objective was to understand the flow of irrigant in a curved canal, only the side view was captured. The needle was captured using an objective lens with a 1.25x magnification, whereas the image was recorded at 10x magnification. The depth of focus of the objective was calculated to be 120 micrometers, which is a comparable approximation to previously validated counts. The recordings were then sent for further analysis using the DaVis 9 software (LaVision).

### Fluid dynamics of irrigants

The ANSYS Workbench 18.2 software facilitated an understanding of the flow of irrigants through CFD, as its finite volume-based solver enables accurate discretization of fluid flow.

The preparation and pre-processing of the models involved converting the STL file into 2D cross-sectional models by slicing the 3D CAD model. The STL file derived the model’s basic outline by replicating the lumen of each needle in the canal space of the double curvature tooth (Supplementary Material). The flow domain was set to each needle’s insertion in accordance with their respective mean DOP in a curved canal. Since all needles could easily cross the first curvature, the flow domain visualized the irrigant flow in the second curvature. At their respective depths, the needles were placed in the root canals as centrally as possible. The external and internal diameters of the needles, as observed under SEM, were accurately replicated in their corresponding geometrical model.

The meshing strategy included a combination of hexahedral and tetrahedral meshes. The density of the meshes was higher in areas where the velocity gradient was expected to be high (needle vents and canal walls). A grid independence check was performed to check the minimum number of cells required to ensure a grid-independent flow simulation. Coarse meshes were first placed, and a preliminary simulation was conducted to identify areas of high volatility. The count of the mesh grid after their refinement to finer sizes varied between 1,792,299 and 2,002,33 (depending on the needle type).

No-slip boundary conditions were applied at the walls of the canals and needle. Furthermore, they were assumed to be smooth, rigid, and impermeable to liquids. The outlet boundaries were set to a zero pressure gradient to simulate the elimination of the debris reintroduction. No symmetrical boundary conditions were assumed along the z-y plane. The irrigant’s flow was visualized as directed from the distal end of the needle towards the orifice of the root canal, where atmospheric pressure was applied. The flow domain was assumed to be filled with irrigant.

A velocity inlet boundary condition was applied at the inlet of the needle. A flat velocity profile was set to 8.6 m/s with a flow rate of 0.26 ml/s through a 30-gauge needle used for positive-pressure irrigation [23,24]. The irrigant was modelled to be an incompressible Newtonian fluid with a density of 1,040 kg/m^3^ and a viscosity of 0.99 × 10^-3^ Pa·s [25]. The operational condition was 9.8 m/s^2^ to mimic the clinical conditions.

ANSYS Fluent (18.2) was used as a finite volume solver. An implicit iterative solver was used to obtain numerical solutions by solving the Reynolds-Averaged Navier-Stokes equation. An unsteady isothermal flow was assumed and no turbulence model was used. To avoid transient effects, steady-state solutions were first obtained for use as initial conditions in unsteady simulations. All transport equations were discretized to ensure second-order accuracy, thereby efficiently capturing jet streams, vortices, and recirculations. The transport equations solved for continuity and momentum facilitated numerical convergence. This criterion was set to 10^-4^ for maximum scaled residuals. Based on the Courant number, an adjusted time step of 2 × 10^-6^ was used with a computational flow time of 10 s.

The parameters of interest computed by the flow fields included flow pattern, wall shear stress, pressure, and velocity magnitude. The wall shear stress was calculated by solving the viscous traction on the wall (viscosity multiplied by velocity gradient). These computations were done in a Windows 11 edition of a 64-bit operating system, paired with an 11th Gen Intel Core i5 processor (2.42 GHz) and 8 GB of RAM. The post-processing operations included visualizing the flow fields in terms of velocity streamlines. These were later compared with the visualizations from the PIV setup, both qualitatively and quantitatively. The latter was done by calculating the correlation coefficient and mean absolute percentage error.

## Results

### In vitro analysis for depth of penetration of the needles

The mean DOP for NTO, SSDV, SSSV, SP were 14.65 mm, 18.85 mm, 17.07 mm, and 20.09 mm, respectively (Table 1). Statistical analysis using ANOVA demonstrated a significant difference in the DOP (*p* < 0.05). Tukey’s post hoc test, conducted for intergroup comparisons, revealed significant differences between all four groups. Thus, DOP of the four needles increased in the following order: NTO < SSSV < SSDV < SP (Table 1).

**Table 1:** Comparison of the depth of penetration between four groups using a one-way ANOVA test.

One-way ANOVA					
Group	*N*	Mean	SD	95% confidence interval for mean	
				Lower bound	Upper bound
I (NTO)	10	14.65	0.085	14.589	14.711
II (SSDV)	10	18.85	0.158	18.737	18.963
III (SSSV)	10	17.07	0.189	16.935	17.205
IV (SP)	10	20.09	0.088	20.027	20.153
Total	**40**	**17.67**	**2.075**	**17.001**	**18.329**
Tukey’s post- hoc analysis					
**Intergroup comparison**		**Mean**	***p*-value**	**95% confidence interval**	
				**Lower bound**	**Upper bound**
I (NTO)	IV (SP)	−5.44	<0.001	−5.606	−5.274
II (SSDV)	I (NTO)	4.2	<0.001	4.034	4.366
	IV (SP)	−1.24	<0.001	−1.406	−1.074
III (SSSV)	II (SSDV)	−1.78	<0.001	−1.946	−1.614
	I (NTO)	2.42	<0.001	2.254	2.586
	IV (SP)	−3.02	<0.001	−3.186	−2.854

SD: standard deviation.

### In silico analysis of wear

Thematic analysis of the two qualitative reports identified three themes that described the wear on the needles: surface roughness, cracks, and fracture. Table 2 summarizes the report’s findings.

**Table 2:** Wear on the needle.

Type of needle	Surface roughness	Cracks	Fracture
NiTi Open-ended	Scratch marks, voids, abrasive wear	-	-
Stainless Steel Double Vented	Shear bands	Crack initiation	Buckling type (occurs due to high axial compressive stress and low ductility of the material)
Stainless Steel Single Vented	Austenite phase grains indicate corrosion resistance	Branching cracks (due to a combination of compressive stress and corrosion)	-
Soft polypropylene	Periodic shedding of the outer layer due to abrasive wear	Not observed due to high elasticity	

**Figure 3:**
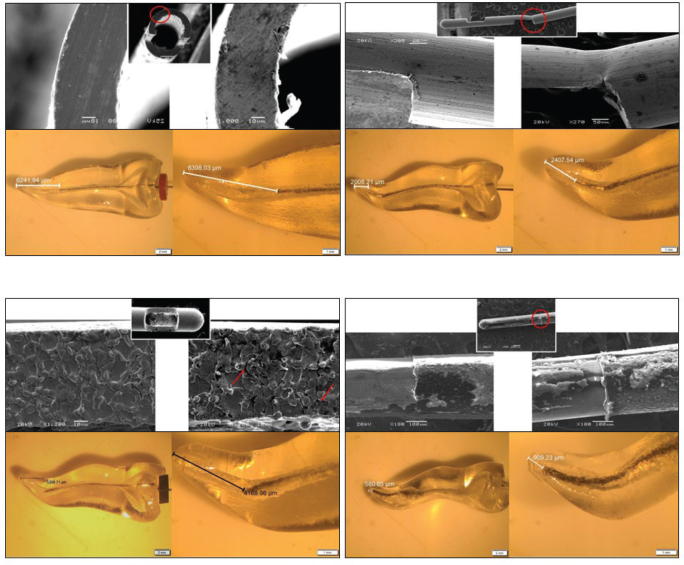
This image depicts the following templates for each needle: (Top) Pre- and Post-irrigation SEM images of wear on the needle with circled areas representing the most common wear change (Bottom) Stereomicroscope calculations for depth of penetration. (Clockwise) NiTi open-ended notched needle, stainless steel double-side vented needle, stainless steel single-side vented needle, soft polypropylene multi-vented needle.

The elasticity of the soft polypropylene needles makes them more resistant to wear. The stainless-steel needles showed minimal abrasive wear and plastic deformation, especially with the SSDV needle. The highest plastic deformation was observed at the junction of the two vents in the SSDV needles, a finding observed in two of the three needles examined under SEM. It was also the site of the highest deformation. A finding observed in both the NiTi open-ended needles and the SSSV needle was cracks that resembled a branched appearance (Figure 3).

### In silico analysis for fluid dynamics

#### CFD model validation

The flow of the irrigant in each case was unsteady, as indicated by the PIV vectors and the CFD colormaps depicting the velocity magnitude. A striking distinction can be observed in that the irrigant flow from the SSSV needle in the PIV imaging revealed the formation of a Moffatt vortex, which was not apparent in the CFD colormap. However, this could be appreciated in the post-processing streamline imaging. Such a discrepancy can be attributed to the representation of scalar magnitudes in colormaps, which contrasts with the trajectory-based representation in streamline imaging.

The flow comparisons of PIV and CFD for the remaining three syringes showed a close agreement in terms of the direction of the fluid flow, axial velocity, location, and size of vortices, as well as the recirculation of the fluid, wherever applicable (Supplementary Material).

**Figure 4:**
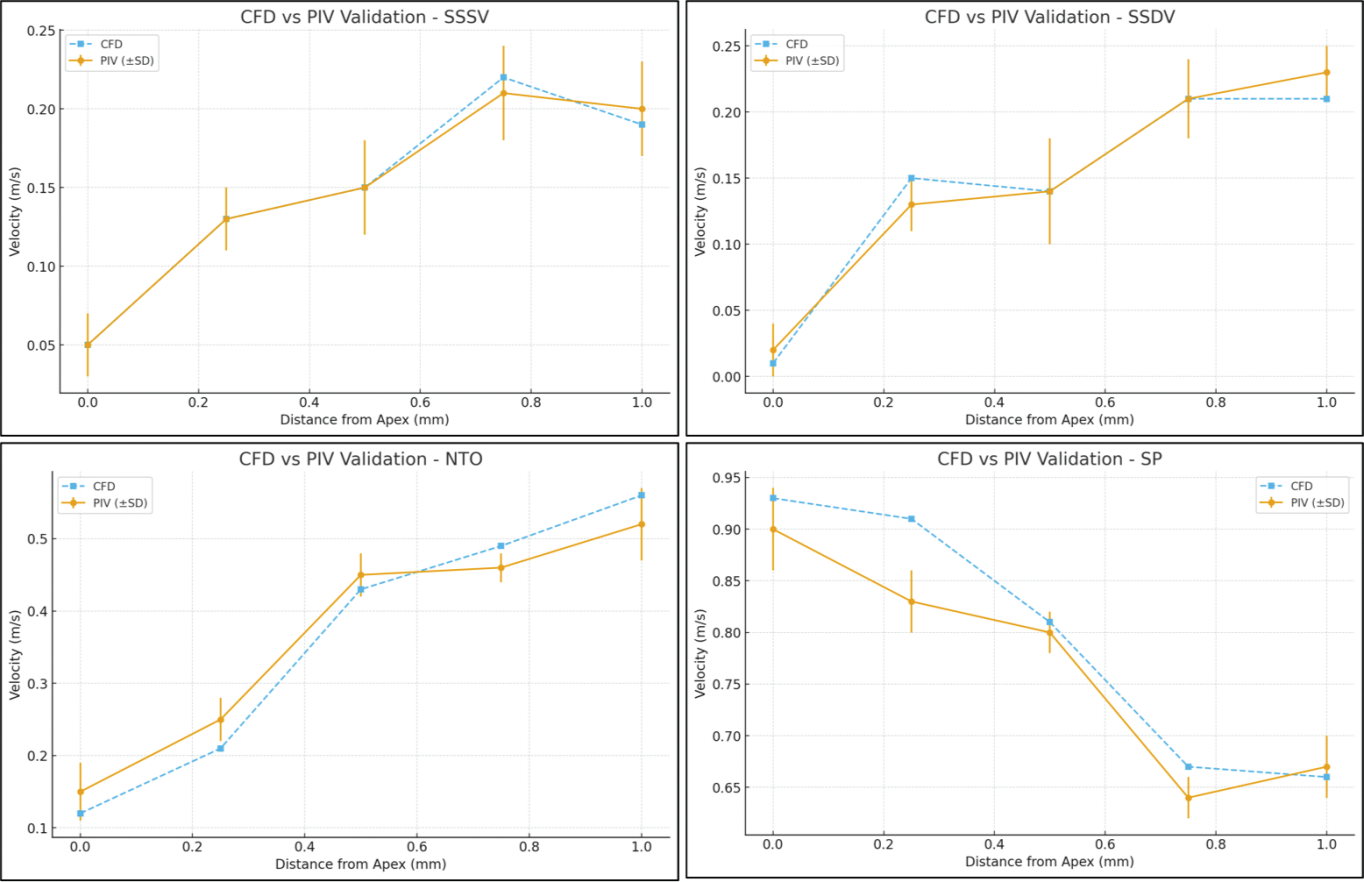
Comparative analysis of axial velocity data derived from computational fluid dynamics and particle image velocimetry for model validation of all four needle designs. SSSV: Stainless steel single-side vented; SSDV: Stainless steel double-side vented; NTO: NiTi Open-ended needle; SP: Soft polypropylene multi-vented needle.

Velocity profiles of each needle in the PIV and CFD images were analyzed in a region 1 mm from the apex at different intervals on the y-axis. A strong agreement was computed between the experimental and the CFD models as the mean absolute percentage error was ≤15% for each needle and the correlation coefficient (*R*^2^) was 0.993 (NTO), 0.967 (SSDV), 0.953 (SSSV), and 0.991 (SP) (Figure 4).

#### Wall shear stress

**Figure 5:**
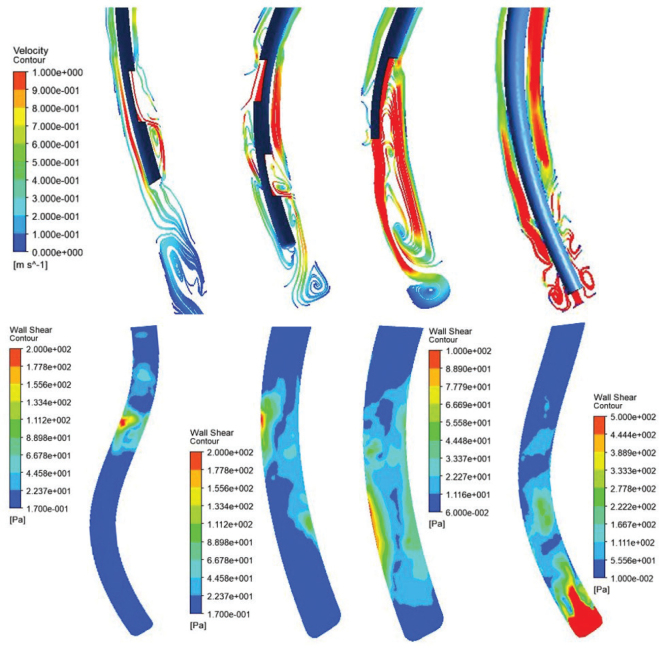
(Top) Streamline depiction of irrigant flow as derived from post-processing for all four needles (from left to right): Stainless steel single-side vented needle, Stainless steel double-side vented needle, NiTi open-ended notched needle, soft polypropylene multi-vented needle. (Bottom) Wall shear stress of each needle at different areas of the root canal. (Clockwise): Stainless steel double-side vented needle, NiTi open-ended notched needle, Stainless Steel Single-side vented needle, soft polypropylene multi-vented needle.

The wall shear stress of NiTi open-ended needles touched a local maxima of 800 Pa when the irrigant made its first contact with the canal wall. A consistent shear stress between 200 and 600 Pa was observed in the middle third of the canal. In the SSDV needle, the two separate streams of jet led to the recording of two independent zones on the canal walls near to the vents wherein the wall shear stress was transiently high. The lowest wall shear stress was observed in the SSSV needle, whose drag force was least pronounced in the apical third. The soft polypropylene needle exhibited the highest wall shear stress in the apical region. The backflow of the irrigant reached the middle third of the canal, recording values between 200 and 300 Pa (Figure 5).

#### Flow profile

The flow profile of the irrigant reached its maximum velocity as it exited the needle lumen from the SSSV needle. A relatively thin jet of irrigant was dispersed after hitting the canal wall facing the vent, after which its axial velocity dropped along with the formation of an annulus in the apical region. Similar features of the irrigant could be observed after it was released from the SSDV needle. However, owing to the release of two separate streams, the velocity contours were faster, and a symmetrical boundary layer along the canal walls could be appreciated. Three zones of irrigant replacement could be observed in the SSDV needle.

In comparison to other needles, the irrigant was released from a higher point from the NiTi needle. Moreover, owing to its open-ended design, a noticeable velocity core was appreciated in the middle third of the canal. The resulting gush in the apical region created visible eddies. In the non-metallic syringe, the recirculation zone could be appreciated at the apical end of the canal due to the formation of high-velocity swirls created by the exit of the irrigant through the multiple side-vents. The velocity profile of the irrigant released through this needle saw the least bypass areas and maximum lateral spread (Figure 5).

## Discussion

An *in vitro* investigation, backed by the precision of *in silico* methods, can help elucidate the biological pathway of irrigants in a complex root canal system. Multiple strategies to enhance the efficacy of irrigation in endodontics have been explored, including modifications to the chemical properties of irrigants and needle designs [11]. To our knowledge, this is the only study that compares metallic and non-metallic needle tips for evaluating FD simulated in a tooth model with double curvature.

Stereolithography printed see-through models of mandibular first premolars using polymethyl methacrylate were used in this study. A single canal in each model had a double curvature configuration, as they were representative of some of the most challenging aspects of endodontics, wherein manual or rotary instruments often fail to reach (cracks, crevices, isthmus, accessory canals, and apical deltas) [11, 26]. Moreover, the transparency of the tooth models made it easy to understand the aspects of endodontic irrigation, which is otherwise a clinically blind procedure.

DOP is a characteristic that influences the extent of irrigant replacement, shear stress on canal walls, and apical pressure. Apical preparation influences both irrigation replacement and DOP. Apical preparations of #30.06 or more have been previously used for root canals to have high irrigant flow and prevent binding of the needle tip [8]. Moreover, a minimum enlargement of 25% has been recommended to ensure optimal coverage of the irrigant [22]. Our study demonstrated that curved canals with apical preparation of #30.04 successfully allowed irrigant flow and adequate penetration of the irrigation needles. A stereomicroscope helped to understand the DOP of different needle designs, as it allows for accurate visualization, is easily reproducible, and eliminates any risk of bias while making quantitative observations [27].

The mean DOP was the least for the open-ended NiTi needles. This finding is in agreement with previous work that has shown that open-ended needles are more difficult to advance in curved canals [8,28]. The non-metallic, multi-vented SP irrigation needle, showed the highest mean DOP, which can be attributed to its flexibility and less plastic deformation.

Wear in brand-new needle tips can be addressed as precursors to wear resistance and cyclic fatigue [29]. Thus, in this study, we attempted to give a new dimension to the tribology of irrigation needles by understanding the wear of brand-new metallic and non-metallic needle tips. Moreover, since irrigation needles are marketed as single-use materials, it is essential to analyze that the wear on their external surfaces (owing to their use in different morphologies of root canals and the influence of different irrigants) does not affect irrigant dynamics [22].

An irrigant’s wall shear stress is a property that defines whether it has sufficient drag force to remove biofilm from the canal walls. Based on the results of our study, no single needle was effective throughout the entire length of the canal. Despite the non-metallic syringe exhibiting a wider span (defined by the contact that an irrigant makes with the canal walls in transverse range), its shear stress was high in the apical region. When coupled with its relatively higher axial velocity near the apex, the irrigant’s flow might cause a risk of apical extrusion of debris. The SSDV needle and NiTi needle in this regard exhibited noticeable vortices in the apical region.

According to Yu et al., a wall shear stress of 100 Pa is considered a threshold for effective removal of smear layer. Based on the results of our study, all needle designs exceeded this range, but not across the entire length of the canal. Moreover, none of the metallic needles displaced an overzealous static pressure in the apical region, thus reducing the risk of apical extrusion of debris. Axial velocity also contributes to apical extrusion of the debris. A velocity of over 0.1 m/s is considered clinically adequate for irrigant replacement [9]. In our study, this velocity was observed in almost every case, except in the apical areas of the vented needles. The axial velocity for the soft polypropylene needle in the apical region was the highest. In the same study by Yu et al., the authors stated that side-vented needles had a higher and more consistent depth of shear stress in canals with a curvature greater than 20° [5]. This could also be observed in our study for SSDV needles, but not for SSSV needles. For SSSV needles, the maximum wall shear stress was observed in the area that was in proximity to the vent. This observation aligns with the one reported by Wang et al., who studied irrigant flow through SSSV in a mandibular molar with a C-shaped canal [30].

Previous studies on the flow of irrigants have made macroscopic observations, such as the displacement rate of a colored dye from the irrigant and the radiodensity of a partly radiopaque solution. However, the significant shortcomings of these methods were that they only described unconcealed events and that radiographs could seldom describe the dynamics of fluid movement [12, 31]. To overcome these limitations, *in silico* analysis through CFD was chosen to study the flow patterns of the irrigants.

Standardized values for understanding irrigant dynamics through CFD have been used in this study. The Reynolds numbers for the irrigant and its flow characteristic were calculated as applicable for a 30-gauge needle [24]. The time step was governed by the Courant number, as recommended by Hu et al. [4]. Based on the calculations of the finite volume solvers, none of the eddies observed in the CFD results had a turbulence intensity and turbulence viscosity ratio that would exceed 100. Moreover, even in a straight canal, axial velocities as high as 0.79 m/s from an SSSV needle did not project any turbulent flow [21]. In each of our analyzed cases, the flow remained laminar, with only marked turbulence observed near the vents in both the CFD and experimental setups. The Reynolds number remained below the critical value of 1,500 in each case; hence, the model was validated for accurately depicting laminar flow. Pereira et al. also concluded that predictions based on a laminar flow model show greater agreement with PIV data [32].

The models used in our study were validated using PIV. It is not uncommon to see CFD model validations through PIV that utilize fluorescent tracer particles to understand the fluid flow. However, the drawback of this technique is that understanding flow largely depends upon the amount of fluorescence exhibited by these particles. Hence, we chose to use a continuous light source for our model validation through PIV.

The SSSV needle was the only needle in which a dead water zone (a low velocity of irrigant flow that does not allow for irrigant replacement) could be appreciated. This observation was also found in an earlier study by Gao Y, who utilized the SSSV needle to examine the irrigant flow in a single-curved canal [33].

This study has noteworthy limitations. Firstly, though the investigators were calibrated, there was a potential for operator bias in terms of DOP due to the transparency of the tooth models. Understanding the FDs in other orthogonal planes with different needle angulations can provide a better dimensional perspective of the irrigant flow in double-curvature canals. Secondly, the surface roughness of root dentin differs from that of the resin used in the 3D-printed tooth models. Although PIV has been used for model validation, when measuring axial velocity, it often compromises its calculations in an interrogation area that lies near the canal walls [22].

Future studies should focus on the wear of non-metallic needle tips over repeated use under a specific loading factor to understand their cyclic fatigue. Apart from the needle’s angulations, the effectiveness of irrigation can also be compared based on various depths of penetration and the needle’s movement during irrigation. In terms of the irrigant flow, the level of turbulence developed within the needle lumen at a standardized barrel pressure should also be investigated. The double curvature model of the root canal can also be used to compare the effectiveness of positive and negative pressure irrigation techniques, or to investigate the FDs upon activation of the irrigant. Each of these comparisons can be further translated by understanding their effectiveness in eliminating microbes.

## Conclusion

This study concludes the following for the four needle designs in a validated CFD model with a double curvature canal:

- Soft polypropylene needles achieved the highest DOP and irrigant replacement with minimal apical shear stress.
- Double-vented stainless steel needles showed a consistent effective shear stress but were prone to deformative wear.
- Open-ended NiTi needles had the shallowest reach but generated the highest number of vortices.
- The single side-vented stainless steel needle was the only one with a dead water zone in the apical region.

## Supplementary Material


