# Supplementary material for: Efficacy of different irrigation needles used in endodontics: an in silico and an in vitro investigation
Source: Biomater Investig Dent. 2025 Dec 17;12:45148. doi: 10.2340/biid.v12.45148 (PMC12742222; doi:10.2340/biid.v12.45148)
Supplement: Supplementary file 1 [file BIiD-12-45148-s1.pdf]

Supplementary material has been published as submitted. It has not been copyedited or typeset by Biomaterial Investigations in Dentistry.

CAD images of each needle within the canal. Clockwise: Stainless steel single side-vented needle, NiTi Open ended notched needle, Stainless steel double side-vented needle, Soft polypropylene Multi-vented needle.

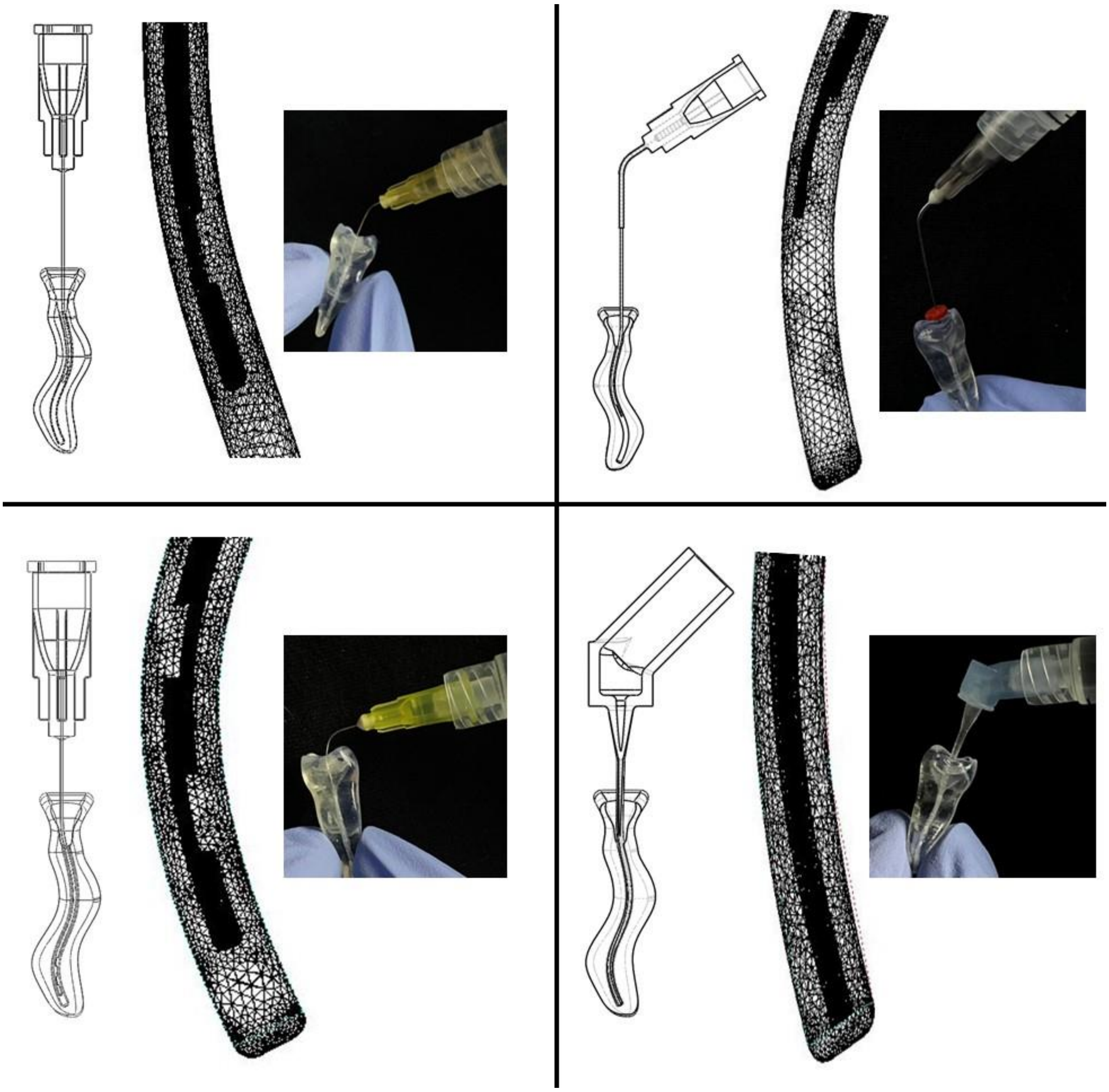

PIV vs CFD for each needle design

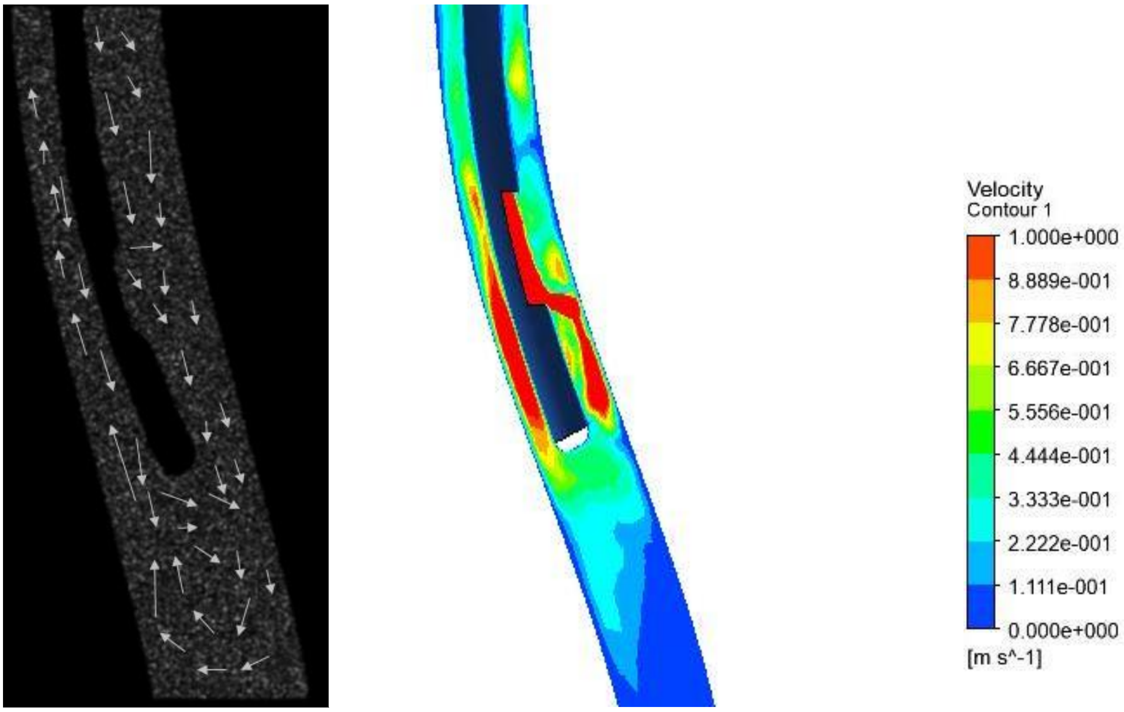

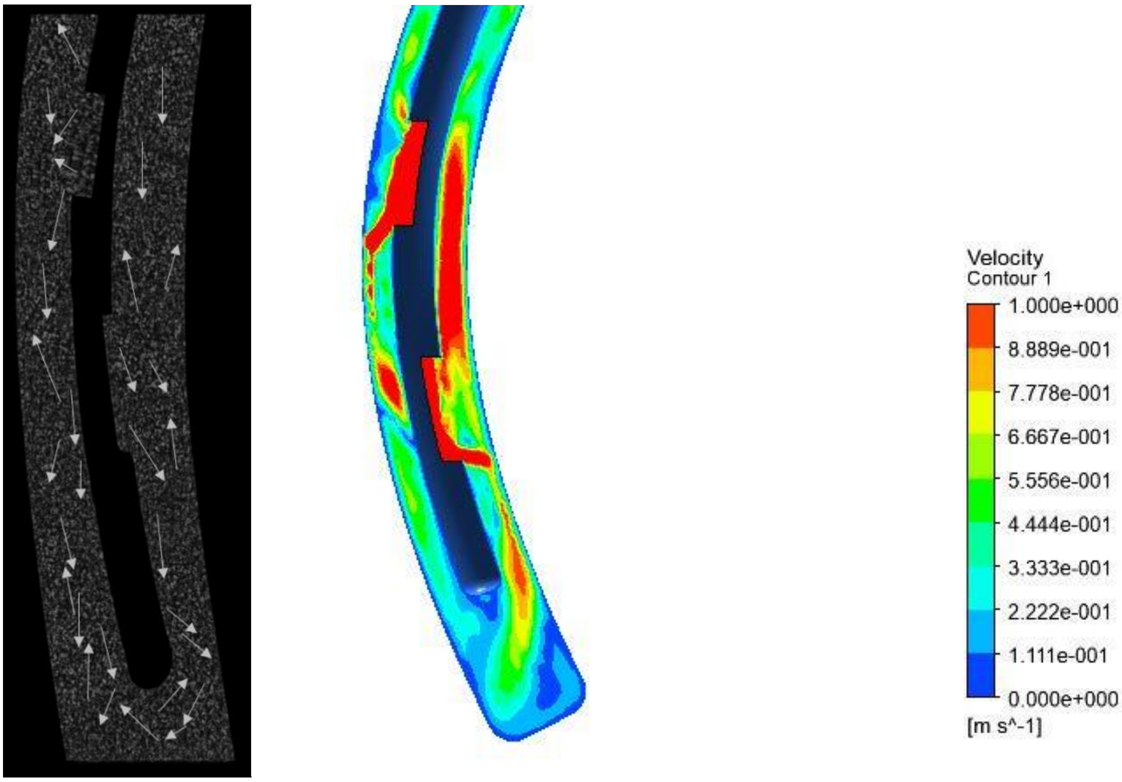
Velocity
Contour 1
1.000e+000
8.889e-001
7.778e-001
6.667e-001
5.556e-001
4.444e-001
3.333e-001
2.222e-001
1.111e-001
0.000e+000
[m s^-1]

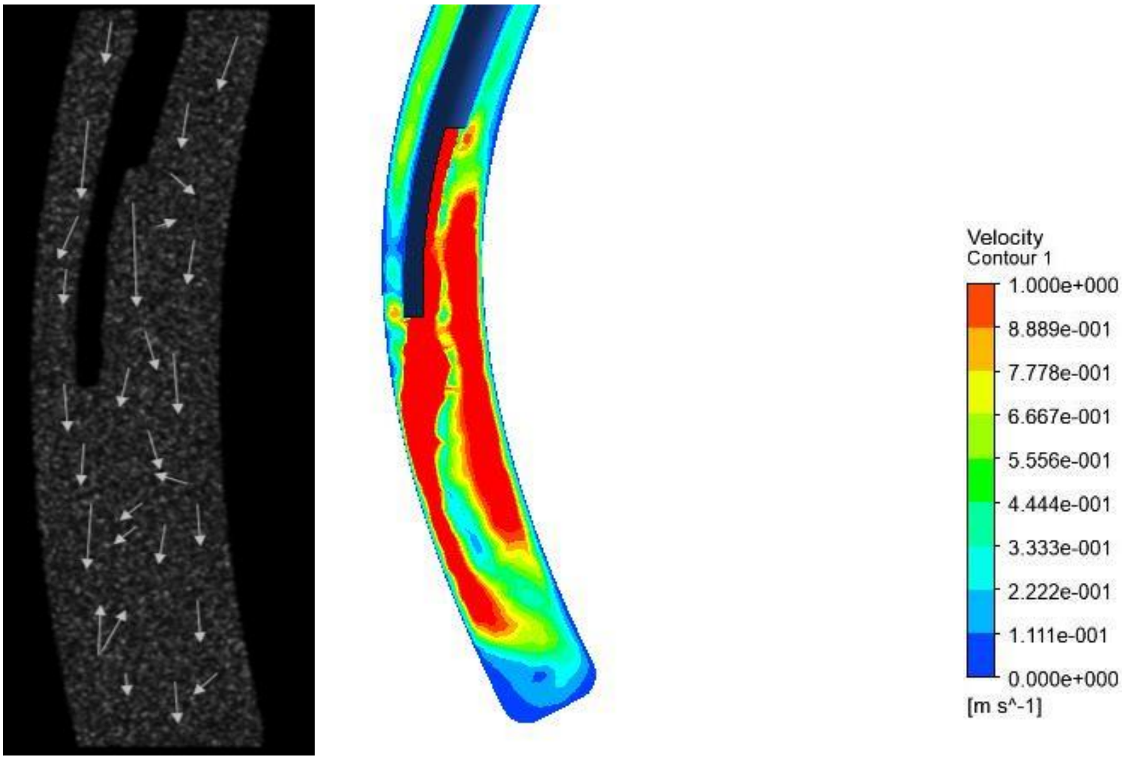
Velocity
Contour 1
1.000e+000
8.889e-001
7.778e-001
6.667e-001
5.556e-001
4.444e-001
3.333e-001
2.222e-001
1.111e-001
0.000e+000
[m s^-1]

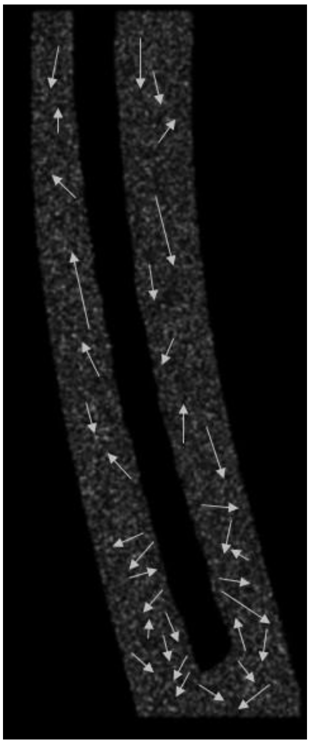

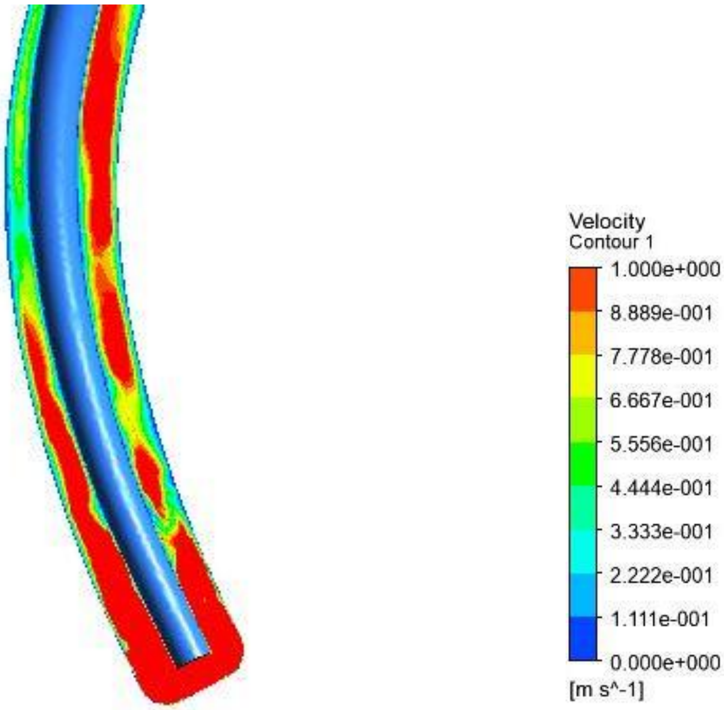
Velocity
Contour 1
1.000e+000
8.889e-001
7.778e-001
6.667e-001
5.556e-001
4.444e-001
3.333e-001
2.222e-001
1.111e-001
0.000e+000
[m s^-1]
